# Impact of endophytic colonization by entomopathogenic fungi on the behavior and life history of the tobacco peach aphid *Myzus persicae* var. *nicotianae*

**DOI:** 10.1371/journal.pone.0273791

**Published:** 2022-09-06

**Authors:** Liesbet Wilberts, József Vuts, John C. Caulfield, Gareth Thomas, Michael A. Birkett, Beatriz Herrera-Malaver, Kevin J. Verstrepen, Islam S. Sobhy, Hans Jacquemyn, Bart Lievens

**Affiliations:** 1 Department of Microbial and Molecular Systems (M2S), CMPG Laboratory for Process Microbial Ecology and Bioinspirational Management (PME&BIM), KU Leuven, Leuven, Belgium; 2 Leuven Plant Institute (LPI), KU Leuven, Leuven, Belgium; 3 Department of Biointeractions and Crop Protection, Rothamsted Research, Harpenden, United Kingdom; 4 Department M2S, CMPG Laboratory of Genetics and Genomics, KU Leuven, Leuven, Belgium; 5 Flanders Institute for Biotechnology (VIB), KU Leuven Center for Microbiology, Leuven, Belgium; 6 Faculty of Agriculture, Department of Plant Protection, Suez Canal University, Ismailia, Egypt; 7 Laboratory of Plant Conservation and Population Biology, Biology Department, KU Leuven, Leuven, Belgium; Gauhati University, INDIA

## Abstract

Entomopathogenic fungi can adopt an endophytic lifestyle and provide protection against insect herbivores and plant pathogens. So far, most studies have focused on *Beauveria bassiana* to increase plant resistance against abiotic and biotic stresses, while only little is known for other entomopathogenic fungi. In this study, we investigated whether root inoculation of sweet pepper (*Capsicum annuum* L.) by the entomopathogenic fungi *Akanthomyces muscarius* ARSEF 5128 and *B*. *bassiana* ARSEF 3097 can improve resistance against the tobacco peach aphid *Myzus persicae* var. *nicotianae*. First, dual-choice experiments were performed to test the hypothesis that the fungi deter aphids via modifying plant volatile profiles. Next, we tested the hypothesis that endophytic colonization negatively affects aphid life history traits, such as fecundity, development and mortality rate. Aphids were significantly attracted to the odor of plants inoculated with *A*. *muscarius* over non-inoculated plants. Plants inoculated with *A*. *muscarius* emitted significantly higher amounts of β-pinene than non-inoculated plants, and significantly higher amounts of indole than *B*. *bassiana-*inoculated and non-inoculated plants. Inoculation with the fungal strains also caused significantly higher emission of terpinolene. Further, both aphid longevity and fecundity were significantly reduced by 18% and 10%, respectively, when feeding on plants inoculated with *A*. *muscarius*, although intrinsic rate of population increase did not differ between inoculated and non-inoculated plants. Sweet pepper plants inoculated with *B*. *bassiana* ARSEF 3097 did not elicit a significant behavioral response nor affected the investigated life history traits. We conclude that endophytic colonization by entomopathogenic fungi has the potential to alter olfactory behavior and performance of *M*. *persicae* var. *nicotianae*, but effects are small and depend on the fungal strain used.

## Introduction

Pest insects pose a major threat to agriculture and horticulture worldwide, causing huge economical losses [1,2]. They can harm crops directly by feeding on plant sap or plant tissues, or indirectly by spreading plant pathogens [1]. Present-day pest control relies heavily on the use of chemical pesticides. However, this involves serious drawbacks such as development of pesticide resistance and potential toxicity to humans and other non-target organisms [3,4]. Therefore, there is an urgent need for an alternative approach that can complement or replace current chemical-based pest management practices. One promising alternative is biological pest control, using natural enemies such as predatory arthropods, parasitoids, or entomopathogenic fungi, bacteria, nematodes or viruses [5,6].

Entomopathogenic fungi represent a large group of fungal species that naturally infect insect populations [7], and several strains have been developed as biocontrol agents [8]. In addition to interacting directly with insect hosts as pathogens, there is growing evidence that entomopathogenic fungi are able to associate with plants [9], often as endophytes [10–13] by colonizing plant tissues without causing symptoms [14]. Some entomopathogenic fungi have been reported as naturally occurring endophytes, but the majority of studies have focused on the introduction of entomopathogenic fungi into plants by artificial inoculation [12,13,15]. In particular, fungal strains from the genus *Beauveria* (Cordycipitaceae) have been successfully established as endophytes in several crops like potato, wheat, cotton, maize, tomato and sweet pepper following artificial inoculation [11,15]. Endophytic colonization by entomopathogenic fungi has been shown to be beneficial for plants as it may increase nutrient uptake or availability, increase tolerance to abiotic stresses, or enhance plant growth [16–19]. Furthermore, an increasing number of studies have reported the potential of endophytic entomopathogenic fungi to increase plant resistance to pathogens [20,21] and insect herbivores [10,12,15,18,22,23]. Although the mechanisms underlying these observations are not yet fully understood, increased protection against insect herbivores may result from enhanced plant defenses and the production of deterrent or toxic molecules produced or induced by the fungi, leading to reduced feeding damage or reduced herbivore performance (e.g. reduced population growth, reduced longevity and fecundity, and prolonged generation time), respectively [21,24–26].

Despite accumulating studies on entomopathogenic fungi helping plants to fend off antagonists, so far most studies have focused on effects of *Beauveria bassiana* to increase plant resistance against biotic and abiotic stresses [22,27–30], whereas only little is known for other entomopathogenic fungi like *Metarhizium* (Clavicipitaceae) and *Akanthomyces* (previously *Verticillium* or *Lecanicillium*; Cordycipitaceae) [15,26,31]. Furthermore, most studies have been performed with sterile substrates, limiting effects of other microorganisms, whereas the use of non-sterile substrates would better approximate natural conditions [15,32].

The aim of this study was to investigate whether endophytic colonization by the entomopathogenic fungi *Akanthomyces muscarius* and *B*. *bassiana* increases plant resistance against aphids, representing one of the major pests in agriculture worldwide [33]. More specifically, we tested the effects of root inoculation of sweet pepper (*Capsicum annuum* L.; Solanaceae) with *A*. *muscarius* ARSEF 5128 and *B*. *bassiana* ARSEF 3097 on the behavior, fecundity, development and mortality rate of the tobacco peach aphid *Myzus persicae* var. *nicotianae* Blackman (Hemiptera: Aphididae). *Myzus persicae* var. *nicotianae* is a subspecies of *M*. *persicae* (Sulzur), one of the most polyphagous aphids, which can damage plants directly through feeding and honeydew deposits, or indirectly by transmitting important viruses [34]. Moreover, the species has developed multiple mechanisms of resistance to almost all major classes of insecticides [35], reinforcing the need for an alternative management approach. First, dual choice experiments were performed to test the hypothesis that the fungi deter the aphids via modifying plant volatile profiles. Next, we tested the hypothesis that endophytic colonization negatively affects key aphid life history parameters, such as fecundity, development and longevity. Experiments were performed with entire plants grown in non-sterile substrates.

## Materials and methods

### Study organisms

Sweet pepper (*Capsicum annuum* L.) cv ‘IDS RZ F1’ (Rijk Zwaan, De Lier, the Netherlands) was used as the focal plant in our study. Plants were grown in a 3:1 mixture of potting mix (Universal potting mix; Agrofino, Ghent, Belgium) and white sand in a plant growth chamber following transplant procedures (see below for details) and watered when required. The growth chamber (MD1400, Snijders Labs, the Netherlands) was set at 23 ± 1°C, 65 ± 2% RH and a 16L:8D photoperiod and was equipped with LED lights to provide a photosynthetic flux density of 790 μmol photons m^-2^ s^-1^. A colony of *Myzus persicae* var. *nicotianae* was obtained from NIOO-KNAW (Wageningen, the Netherlands) and maintained on sweet pepper at 23 ± 1°C, 70 ± 2% RH and a 16L:8D photoperiodic regime. Weekly, fresh plants were provided to support the colony. Two entomopathogenic fungi, *Akanthomyces muscarius* ARSEF 5128 (Ve-6; previously classified as *Lecanicillium muscarium*) and *Beauveria bassiana* ARSEF 3097 (ATCC 74040), were used in this study. Both strains are the active ingredient in commercial bioinsecticides, i.e. Mycotal^®^ and Naturalis^®^, respectively, and were obtained from the Agricultural Research Service Collection of Entomopathogenic Fungal Cultures (ARSEF; New York, USA). *Akanthomyces muscarius* ARSEF 5128 was originally isolated from a greenhouse whitefly in Littlehampton in West Sussex [36]. *Beauveria bassiana* ARSEF 3097 was originally isolated from a boll weevil cadaver in the Rio Grande Valley of Texas [37]. The endophytic capability of this strain has been shown previously when inoculated onto sweet pepper, grapevine and tomato, as its ability to deter or cause mortality of sap-sucking insects when endophytic [27,30,38]. Before use in our study, both strains were inoculated on sweet pepper and reisolated from the leaves. Fungal strains were then stored as agar plugs in glycerol at -80°C until further use.

### Fungal spore suspensions

Stock cultures were plated onto quarter-strength (¼) Sabouraud dextrose agar supplemented with yeast extract (Oxoid Holdings Ltd, United Kingdom) (SDAY), and transferred to the same agar medium once again before use. Next, fungal strains were cultured on SDAY for seven days at 25°C in darkness. Conidial suspensions were then prepared by gently scraping conidia from the dishes after flooding the plates with sterile physiological water. The resulting suspension was filtered through microcloth (Mira Cloth, Merck, Massachusetts, USA) to remove hyphal fragments. After homogenizing the suspension, conidial concentration was determined under the microscope with a Bürker hemocytometer and adjusted to 1×10^7^ spores mL^-1^. Prior to experimentation, conidial viability was checked by plating a 100 μL aliquot of 1×10^3^ spores mL^-1^ on three SDAY plates and counting the number of germinated and ungerminated conidia under the microscope after 24 h of incubation at 25°C. Conidia were considered germinated when the size of the germ tube was two times longer than the diameter of a conidium. The germination tests showed >90% viability rate for all experimental fungal spore suspensions used.

### Plant inoculation and verification of endophytic colonization

For each experiment, plants were inoculated at the first-true leaf stage by first rinsing the roots of the seedlings under running tap water, and subsequently submerging them in 10 mL of conidial spore suspension. Preliminary experiments showed that this inoculation procedure consistently resulted in successful endophytic establishment of the studied entomopathogenic fungi in sweet pepper. Additionally, roots of another set of seedlings were submerged in 10 mL physiological water to be included as non-inoculated (control) plants. Subsequently, seedlings were transplanted in 10.5 cm diameter plastic pots and maintained, under the same environmental conditions as mentioned earlier, for four weeks in the growth chamber before use in the experiments. At that stage, plants were around 15 cm high with 7–8 fully expanded leaves. Endophytic colonization was assessed by PCR using the species-specific primer combinations ITS1F [39] and Am_Rv1 (5’-AGATGCTGATAATACAGAGTT-3’) and ITS1F and Bb_Rv1 (5’-GATGCTGGAATACAAGAGTTTGAG-3’) to detect *A*. *muscarius* and *B*. *bassiana*, respectively. While ITS1F is a universal fungal primer [39], reverse primers were designed to be species-specific. Specifically, the fifth true leaf of both inoculated and control plants was sampled at the end of all experiments, except for the life history assay where it was collected after all adult aphids were removed from the plant (see section Life history). For each experiment, genomic DNA was extracted from the leaves of ten plants per treatment (except for the experiment when volatiles were collected, where nine plants were tested) as described previously [40] and subjected to PCR amplification. Amplification (1 μL DNA) was carried out in a 20 μL reaction volume using 1 unit Titanium Taq DNA polymerase (Clontech Laboratories, Palo Alto, CA, USA) according to the manufacturer’s protocol. Before amplification, samples were pre-heated to 94°C for 2 min, followed by a cycling protocol of 35 cycles consisting of 45 s at 94°C, 45 s at 63°C (*A*. *muscarius*) or 61°C (*B*. *bassiana*) and 45 s at 72°C, with a final 10 min extension step at 72°C. Evaluation of the specificity of the primer sets against various fungi, including the target species as well as a number of close relatives, revealed that both primer combinations were species-specific under these conditions. Samples were analyzed by electrophoresis through 1.5% agarose gels stained with ethidium bromide and DNA was visualized with UV in an InGenius 3 gel imager (SyngeneTM, Cambridge, UK). For a number of samples, the identity of the fungi was verified by sequencing the amplicons with the reverse primer used for the PCR. To rule out the possibility that the fungi were on the outside of the leaves instead of the inside, the adaxial and abaxial side of the fifth true leaf of a number of plants was pressed onto Potato Dextrose Agar (PDA; Holdings Ltd, United Kingdom) plates and incubated for three days at 25°C. Likewise, the wash solution of some washed leaves was plated, and a number of washed, surface-sterilized leaves was plated to confirm fungal presence in the leaves. Obtained fungi were identified by sequencing the internal transcribed spacer (ITS) region after PCR amplification with the universal primers ITS1F and ITS4 [39].

### Two-choice Y-tube assay

A Y-tube olfactometer was used to determine aphid responses to volatiles emitted from fungus-inoculated sweet pepper plants against non-inoculated plants. On one side of the olfactometer, an individual inoculated plant was put in a plastic container (height: 23 cm; diameter: 10 cm). On the other side, a non-inoculated control plant was placed in an identical container. Plant pots were wrapped with aluminum foil to avoid interference with soil volatiles. Charcoal-filtered air was pumped into the containers and driven out the containers again through plastic tubes at a flow rate of 2 L min^-1^. Air outlets from each container were connected to the arms of the Y-tube (stem: 20 cm; arms: 12 cm with a 60° angle at the Y-junction; inner diameter: 1.5 cm). The Y-tube was positioned on a table and mounted at a 20° incline to stimulate the aphids to move towards the bifurcation. The Y-tube was homogeneously illuminated by four 24 W T5 TL-fluorescent tubes (16 × 549 mm, 1350 Lumen, 5500 K) from a height of 45 cm and enclosed in white curtains to avoid visual interference from the surrounding space. Bioassays were performed with a total of 120 winged aphids per choice experiment, which we starved for 1 h prior to the experiment. Aphids were tested in cohorts of five (*n* = 24) that were released at the basis of the Y-tube. Olfactory response was evaluated 20 min after release. Trial runs indicated that a 20-min time was adequate for the aphids to make a choice. Individuals that had passed a set line in one of the arms (1 cm from the junction) at the time of evaluation were considered to have made a choice. Aphids that did not pass the line were considered non-responders and were eliminated from statistical analysis. For every run, new aphids and new plants in cleaned containers were used. Additionally, the Y-tube was renewed after six releases, and the arms were flipped 180° to minimize any spatial effects. The assay was also performed with two non-inoculated plants (*n* = 24) to confirm that the aphids showed no preference for either olfactometer arm. At the end of the assay, all olfactometer parts were rinsed with tap water, distilled water, acetone and pentane, after which the parts were kept for 10 h at 150°C. All bioassays were conducted at 23 ± 2°C, 65 ± 5% RH, and were performed between 09:00 and 18:00. Experiments were set up randomized over several days.

### Two-choice arena assay

To investigate whether aphid olfactory responses are modified when aphids can make visual and physical contact with the plants, a second type of bioassay was performed. To this end, plants were first laid on their side, and 4 cm of the fifth true leaf of a non-inoculated control plant and the fifth true leaf of an inoculated plant were fed through a slit in the short sides of a rectangular Petri dish (9 cm × 12.5 cm × 1.5 cm) (S1 Fig, Supporting Information). Leaves were fixed with a droplet of agar on their adaxial side at the bottom of the plate. Next, the plate was flipped so that the leaves regained their natural position, and the aphids were allowed to walk on the abaxial side of the leaves. For each test, ten apterous adults, starved for 1 h, were released in the middle of the arena, after which the arena was sealed with parafilm to prevent aphids from escaping. Plants were incubated at 23 ± 2°C, 65 ± 5% RH, and a 16L:8D photoperiod condition, provided by four 24 W T5 TL-fluorescent tubes (16 × 549 mm, 1350 Lumen, 5500 K) from a height of 50 cm. Aphid response was evaluated 4 h, 8 h and 24 h (i.e. 2 h after the lights were switched on) after their release by counting the number of aphids on each of the leaves. The assay was also performed with two non-inoculated plants. To avoid spatial effects, plates were randomly oriented with the fungal-inoculated plant on the left side for half of the plates, and the right side for the other half. The experiment was repeated ten times, at two independent time points (*n* = 20).

### Collection and chemical analysis of plant volatile organic compounds

To determine differences in the composition of volatile organic compounds (VOCs) of fungus-inoculated sweet pepper plants and non-inoculated control plants, volatiles were collected by dynamic headspace sampling (air entrainment). Individual sweet pepper plants (*n* = 9) were placed in a glass dome (height: 20 cm; diameter: 23 cm), which was closed with aluminum plates around the stem right above the germ leaves, without constricting the plant. To maintain a positive pressure, charcoal-filtered air at 700 mL min^-1^ was pumped into each dome and drawn out at 600 mL min^-1^ through a collection filter containing Porapak Q (50 mg, 50–80 mesh; Supelco, Merck KGaA, Germany), held between two silanized glass wool plugs in a glass tube (outer diameter: 5 mm). The Porapak Q was conditioned by washing with 1.5 mL diethyl ether (Acros Organics, Thermo Fisher Scientific, USA) and heating at 132°C under a constant stream of purified nitrogen for 2 h. Collections were carried out under laboratory conditions (23 ± 2°C; 65 ± 5% RH; 16L:8D photoperiod) for a period of 48 h. Volatiles were then eluted from the Porapak Q with 750 μL diethyl ether, and were stored at -20°C in ampoules sealed under a stream of purified nitrogen. After volatile collections, the glass domes were cleaned with acetone and baked for 2 h at 175°C before using them in the next set of VOC collections. Before subjecting the samples to chemical analysis, VOC samples were concentrated to 100 μL under a stream of purified nitrogen.

Volatile extracts were analyzed on a gas chromatograph (GC) (Agilent Technologies, 6890 N), equipped with a flame-ionization detector (FID) and a HP-1 capillary column (50 m × 0.32 mm inner diameter, 0.52 μm film thickness). The oven temperature was maintained at 30°C for 1 min and programmed at 5°C/min to 150°C, where it was held for 0.1 min, then at 10°C/min to 230°C and held for 27 min. The carrier gas was hydrogen. Manually, 4.2 μL of sample was injected into the cool on-column injection port of the equipment. Quantification of compounds was achieved by the single-point external standard method with a series of C7-C22 alkanes, where the amount of an analyte was estimated using the peak area of the nearest alkane peak, the amount of which was known. Coupled GC-mass spectrometry (GC-MS) analysis of eluted volatiles was performed using a Waters GCT Premier-TOF mass spectrometer (mass range 40–550 a.m.u., ion source temperature 200°C), coupled to a GC fitted with a DB-5 capillary column (30 m × 0.25 mm inner diameter, 0.25 μm film thickness; oven temperature was maintained at 50°C for 2 min and programmed at 5°C/min to 180°C, then immediately at 20°C/min to 270°C and held for 5 min), with a heated (250°C) inlet (split ratio 1:5) and helium as carrier gas (1 mL/min). Tentative identifications were made by comparison of mass spectra with NIST 2005 mass spectral database. Confirmation of peak identity was made by comparison of their Kováts index (KI) values and GC peak enhancement with authentic compounds. (*RS*)-β-Pinene (>95%), myrcene (>90%), (*E*)-caryophyllene (98%) and heptadecane (99%) were purchased from Sigma-Aldrich (UK); terpinolene (90%) was obtained from Fluka (UK), and indole (99%) was from Avocado Research Chemicals, (UK). (*E*)-Ocimene was synthesized as previously described [41].

### Life history

The development and survival of *M*. *persicae* var. *nicotianae* was assessed by confining ten wingless adult aphids in a clip cage (inner diameter: 3.5 cm) on the fifth true leaf of either inoculated or non-inoculated plants. Aphids were then allowed to reproduce for 48 h, after which the adults were removed and 25 one-day old nymphs were kept in each clip cage. In order to determine nymphal development time, we counted the number of nymphs on a daily basis until they reached adulthood, whilst recording and removing dead nymphs to assess mortality rates. Aphids were considered adults when they produced their first offspring. At that time, adult aphids and offspring were removed from the clip cage. Likewise, the number of winged and wingless adults was recorded. Among these, one apterous adult was transferred to a new clip cage on the sixth true leaf of the same plant to monitor reproduction. At the same time, the fifth leaf was sampled for verification of endophytic colonization. The number of offspring was recorded daily, while at the same time nymphs were removed until the death of the parent aphid. The experiment was performed at a different time for each fungus (and control) using ten plants per treatment and was replicated twice in time (*n* = 20). All experiments were performed in a growth chamber at 23 ± 1°C, 65 ± 2% RH and a 16L:8D photoperiod. Altogether, the following parameters were recorded: percentage of nymphal mortality, development time (from birth until first reproduction), adult morph ratio, fecundity and adult longevity (from onset of reproduction until death). Fecundity was determined based on the total number of offspring produced per aphid and by the number of offspring produced per day. Furthermore, the intrinsic rate of natural population increase (rm) was calculated using the formula of Wyatt and White (1977) [42]:

$$rm=0.738(\frac{\text{ln}(FD)}{D})$$

In this formula, the natural logarithmic number of nymphs produced over a time equivalent to the development time starting at the production of the first nymph (FD) is divided by the development time (D), and multiplied by a correction factor obtained from the mean pre-reproductive times (time between adult moult and onset of reproduction) for diverse aphid species (0.738). Each plant with aphids served as a replicate, giving a total of 20 replicates.

### Statistical analyses

Aphid olfactory response in the Y-tube assays was analyzed using a Generalized Linear Mixed Model (GLMM) based on a binomial distribution with a logit link function (logistic regression) using inoculation treatment as a fixed factor (performed with the ‘glmer’ function from the lme4 package) [43]. Each release of one cohort of five individuals served as a replicate, giving a total of 24 replicates. To adjust for overdispersion and to prevent pseudoreplication, the release of each cohort was included in the model as a random factor. The number of aphids choosing the control or treatment side in each cohort was entered as response variable. To examine the preference of the aphids, we tested the null hypothesis (H_0_) that aphids showed no preference for any olfactometer arm (i.e. 50:50 response) by testing H_0_: logit = 0, which equals a 50:50 distribution. Similarly, data obtained in the two-choice arena assay were analyzed using a GLMM with plant treatment and evaluation time as fixed factors. For this analysis, each arena with one cohort of ten individuals served as a replicate, giving a total of 20 replicates, which were included in the model as a random factor.

Differences in plant volatile composition between the different treatments were visualized by a principal component analysis (PCA) using the concentrations of the detected volatiles as dependent variables. Additionally, a non-parametric multivariate analysis of variance (perMANOVA) was used to investigate whether the VOC blends differed between the different fungal treatments and the non-inoculated plants. We performed 1000 permutations to assess the significance of the observed pseudo *F*-statistic. The perMANOVA was performed using the adonis2 function of the vegan package [44] in R. This analysis was followed by a post hoc pairwise comparison (with pairwise.adonis2 function). Differences between individual plant volatiles (ng/h/g fresh plant weight) per treatment were analyzed using ANOVA and Fisher`s LSD test on log-transformed values.

Nymphal mortality and adult morph ratio were analyzed by means of a GLMM based on a binomial distribution with a logit link function using plant treatment as fixed factor, and plant as random factor. Nymphal development time, adult longevity and total number of offspring were analyzed using a GLMM based on a Poisson distribution with a log link function using plant treatment as fixed factor and plant as random factor. The intrinsic rate of natural aphid population increase was analyzed using a GLM based on a Gamma distribution with an inverse link function using plant treatment as fixed factor. The number of offspring per day was analyzed by means of a full factorial GLMM based on a Poisson distribution with a log link function using plant treatment and day as fixed factors. The plant was included in the model as a random factor and the number of offspring per day was entered as response variable. An analysis of variance Type III test was performed on all models to determine if there was an overall difference between the different treatments. The analysis was followed by a post hoc pairwise comparison (with estimated marginal means using the emmeans package). As the life history experiments were performed separately for each fungus, the statistical analysis was also performed separately for each fungus.

A significance level of α = 0.05 was used to determine significant differences, and results were visualized using the ggplot2 package. All analyses and visualization of the data were performed in R version 3.6.1 [45].

## Results

### Endophytic colonization

In all experiments, endophytic colonization by *A*. *muscarius* ARSEF 5128 and *B*. *bassiana* ARSEF 3097 was assessed by subjecting a sample from the fifth true leaf from ten of the tested plants (when available) to PCR analysis. In total, 79.5% and 76.9% of sweet pepper plants were tested positive when inoculated with *A*. *muscarius* and *B*. *bassiana*, respectively, while the fungi were not detected in non-inoculated control plants (S1 Table, Supporting Information). The identity of a number of PCR amplicons was confirmed by sequencing, reinforcing the specificity of the assays. Incubating imprints or wash solutions of the fifth true leaf of a number plants onto PDA consistently resulted in no detection of the inoculated fungi, confirming their absence on the outside of the leaves. Furthermore, plating of a few washed, surface-sterilized leaves demonstrated fungal presence in the leaves.

### Behavioral response

**Fig 1:**
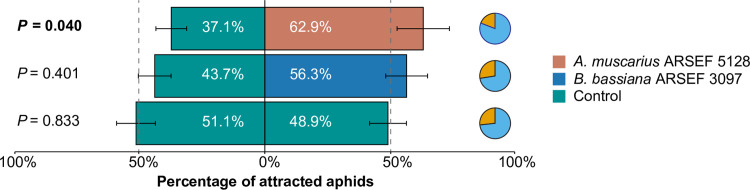
Olfactory response (attraction % ± SEM) of winged *Myzus persicae* var. *nicotianae* when given the choice between sweet pepper plants inoculated with *Akanthomyces muscarius* ARSEF 5128 (orange) or *Beauveria bassiana* ARSEF 3097 (blue) and control plants (green), in a Y-tube olfactometer bioassay (tested in 24 cohorts of 5 adults). *P* values in bold indicate significant differences in aphid response (*P* ≤ 0.05) when compared to a theoretical 50:50 distribution (Generalized Linear Mixed Model). Dashed lines indicate the 50% threshold. Pie charts show the percentage of responding (blue) and non-responding (orange) aphids. Overall aphid responsiveness was 75.6%.

**Fig 2:**
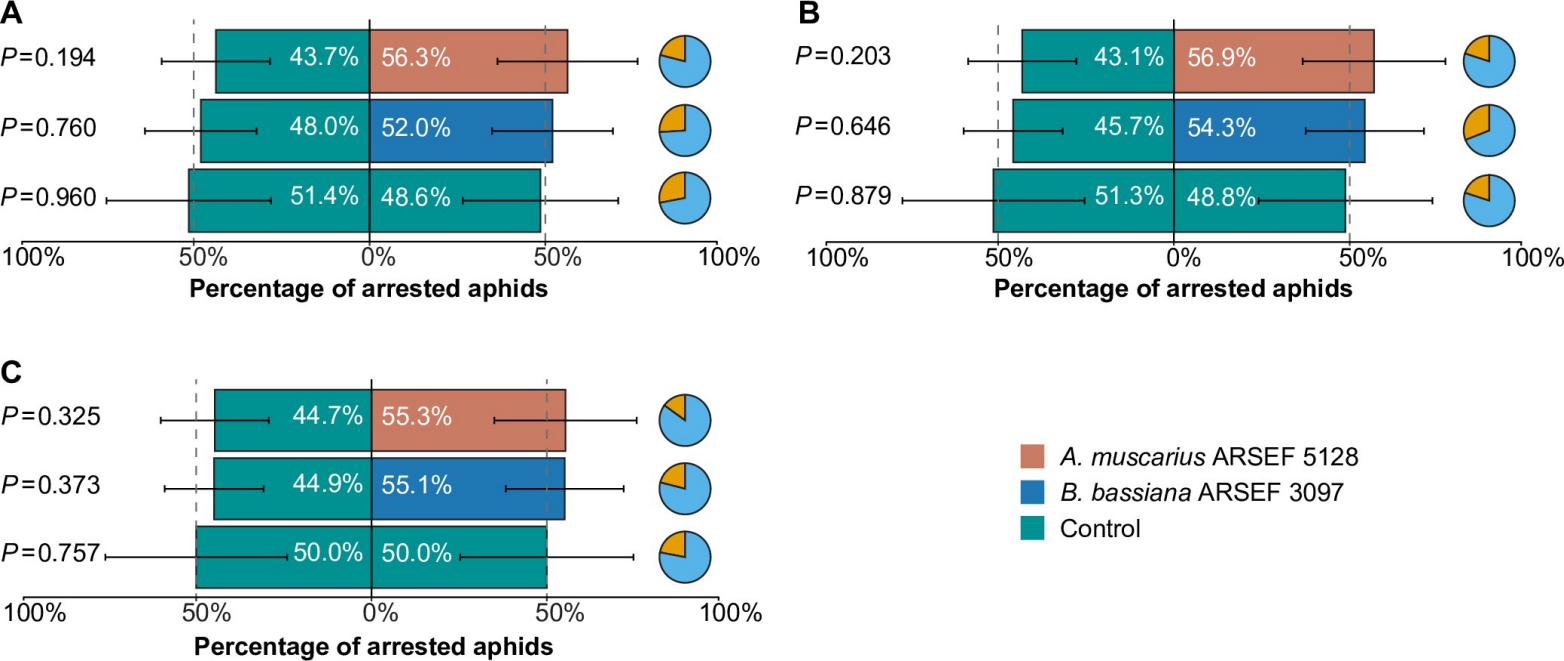
Behavioral response (attraction % ± SEM) of apterous *Myzus persicae* var. *nicotianae* when given the choice between sweet pepper plants inoculated with *Akanthomyces muscarius* ARSEF 5128 (orange) or *Beauveria bassiana* ARSEF 3097 (blue) and control plants (green), in a two-choice arena bioassay (tested in 20 cohorts of 10 adults for the fungal inoculated versus control assay, or 10 cohorts of 10 adults for the control versus control assay). Insect response was evaluated 4 (A), 8 (B) and 24 hours after aphid release (C). *P* values indicate differences in aphid response (*P* ≤ 0.05) when compared to a theoretical 50:50 distribution (Generalized Linear Mixed Model). Dashed lines indicate the 50% threshold. Pie charts show the percentage of responding (blue) and non-responding (orange) aphids. Overall aphid responsiveness was 77.3%.

Aphids showed a significant preference for volatiles emitted by plants inoculated with *A*. *muscarius* ARSEF 5128 over non-inoculated control plants (*P* = 0.040). In total, 62.9% of the aphids preferred the treatment with *A*. *muscarius*, while 37.1% preferred the control treatment. By contrast, no significant difference was observed for *B*. *bassiana* ARSEF 3097 (*P* = 0.401), although inoculation of the fungus attracted 56.3% of the aphids (Fig 1). When aphids could make visual and physical contact with the plants, no statistical differences in preference were found, irrespective of the time of evaluation (4 h: *A*. *muscarius*, *P* = 0.344; *B*. *bassiana*, *P* = 0.665; 8 h: *A*. *muscarius*, *P* = 0.329; *B*. *bassiana*, *P* = 0.646; 24 h: *A*. *muscarius*, *P* = 0.295; *B*. *bassiana*, *P* = 0.596) (Fig 2). In both experiments, an approximate 50:50 distribution was obtained when aphids were subjected either to the volatiles or leaves of two control plants, demonstrating that experimental conditions were met to obtain robust data (Figs 1 and 2). Overall responsiveness of the aphids in the Y-tube assays and arena assays was 75.6% and 77.3%, respectively.

### Chemical analysis of volatile organic compounds

**Fig 3:**
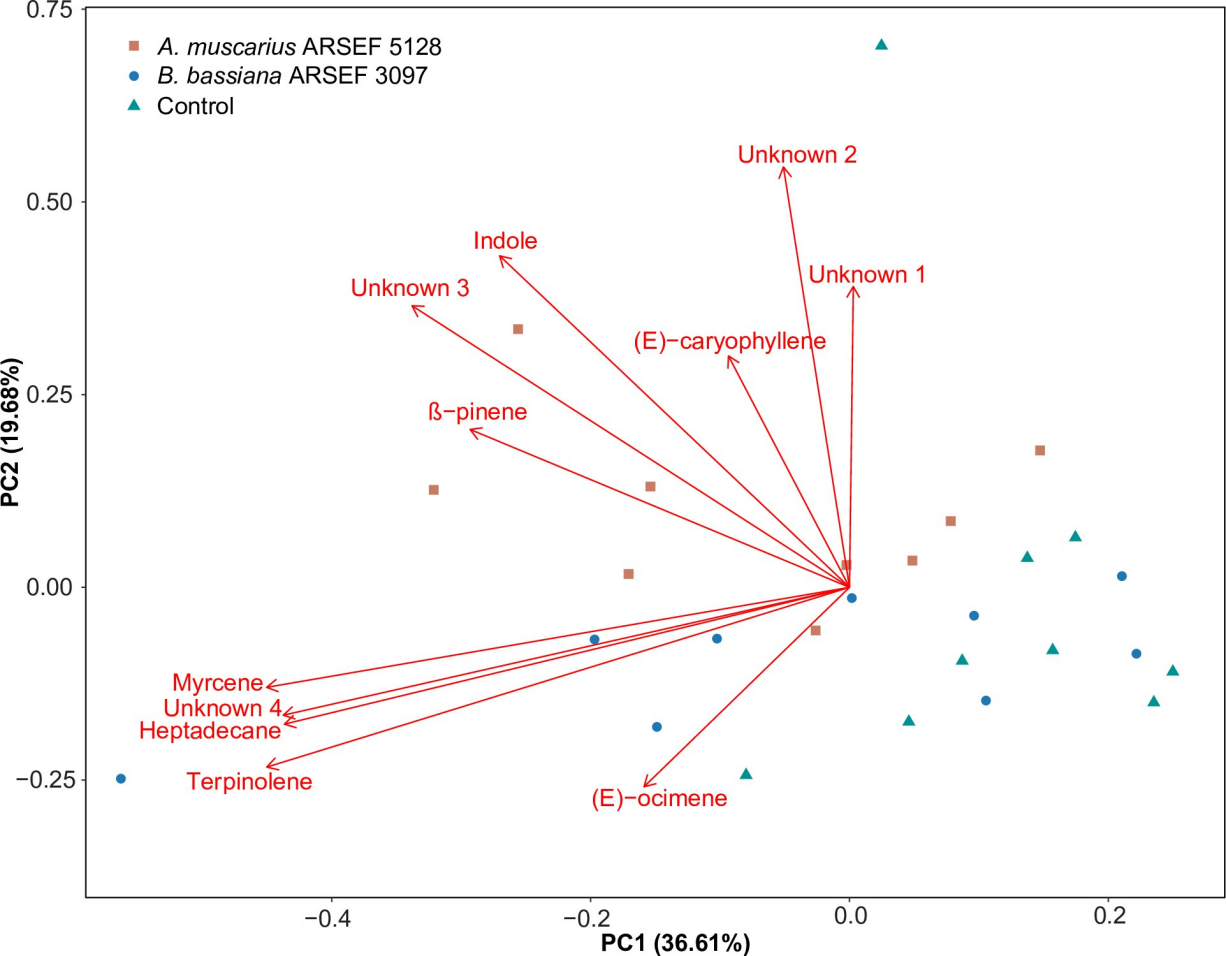
Principal component analysis (PCA) of plant volatiles emitted by sweet pepper plants inoculated with *Akanthomyces muscarius* ARSEF 5128 (orange) or *Beauveria bassiana* ARSEF 3097 (blue) or non-inoculated plants (green) (*n* = 9). Volatiles were collected by dynamic headspace sampling for 48 h and identified by GC-FID/GC-MS. The plot visualizes the location of each analyzed sample on each PC with the percentage of explained variation in parentheses, whereas vectors (in red) visualize the loadings for each variable. Volatile composition differed significantly among treatments (*F* = 2.21, *P* = 0.035; perMANOVA).

The GC-FID/GC-MS analysis revealed a total of 11 compounds, including (*E*)-caryophyllene, heptadecane, indole, myrcene, (*E*)-ocimene, β-pinene, terpinolene and four unidentified terpenes. The principal component analysis (PCA) showed a clear separation between the VOC composition of the *A*. *muscarius-*inoculated plants and the non-inoculated plants. There was no separation between *B*. *bassiana-*inoculated plants and control plants (Fig 3). The first principal component (PC1) accounted for 36.6% of the total variation, the second component (PC2) for 19.7%. perMANOVA revealed that the VOC compositions of the different treatments were overall significantly different (*F* = 2.21, *P* = 0.035). The VOC composition of plants inoculated with *A*. *muscarius* differed significantly from control plants (*P* = 0.011), but not from plants inoculated with *B*. *bassiana* (*P* = 0.350). The VOC composition of *B*. *bassiana*-inoculated plants was also not significantly different from control plants (*P* = 0.112). Sweet pepper plants inoculated with *A*. *muscarius* ARSEF 5128 emitted significantly higher amounts of β-pinene (*P* = 0.003) than non-inoculated plants, and significantly higher amounts of indole than *B*. *bassiana-*inoculated (*P* = 0.008) and non-inoculated plants (*P* = 0.001). Inoculation with either *A*. *muscarius* or *B*. *bassiana* caused significantly higher emission of terpinolene compared to the control (*A*. *muscarius*: *P* = 0.013; *B*. *bassiana*: *P* = 0.017). No significant differences among treatments were found for the other detected compounds (Table 1).

**Table 1:** Amounts^1^ of volatile compounds identified by GC-FID/GC-MS analysis from the headspace of sweet pepper plants inoculated with *Akanthomyces muscarius* ARSEF 5128 or *Beauveria bassiana* ARSEF 3097, compared to non-inoculated plants.

Compound	KI^2^	*A*. *muscarius* ARSEF 5128	*B*. *bassiana* ARSEF 3097	Non-inoculated	P value^3^
β-pinene	972	0.064 ± 0.014 b	0.040 ± 0.006 ab	0.026 ± 0.005 a	0.010
Myrcene	984	0.182 ± 0.035	0.206 ± 0.038	0.107 ± 0.018	0.142
(E)-ocimene	1041	0.135 ± 0.019	0.557 ± 0.249	0.902 ± 0.653	0.706
Terpinolene	1087	3.358 ± 0.369 b	3.913 ± 1.052 b	1.463 ± 0.328 a	0.020
Indole	1261	0.115 ± 0.027 b	0.039 ± 0.004 a	0.043 ± 0.017 a	0.003
(E)-caryophyllene	1434	0.323 ± 0.063	0.215 ± 0.058	0.202 ± 0.082	0.137
Heptadecane	1699	0.637 ±0.119	0.544 ± 0.115	0.383 ± 0.093	0.206
Unknown1^4^	1101	0.051 ± 0.012	0.032 ± 0.006	0.035 ± 0.009	0.418
Unknown2^4^	1367	0.058 ± 0.017	0.031 ± 0.009	0.137 ± 0.127	0.204
Unknown3^4^	1440	0.178 ± 0.024	0.144 ± 0.026	0.124 ± 0.034	0.160
Unknown4^4^	1521	0.254 ± 0.040	0.407 ± 0.145	0.132 ± 0.034	0.119

^1^Average obtained from nine plants ± SEM (ng/h/g fresh plant weight).

^2^The KI (Kováts index) values were obtained on a non-polar HP-1 GC column.

^3^*P* values are from ANOVA (df = 2, α = 0.05) on log-transformed data. Different letters within each row indicate significant differences by Fisher`s LSD post hoc test.

^4^Unidentified terpene.

### Life history

**Fig 4:**
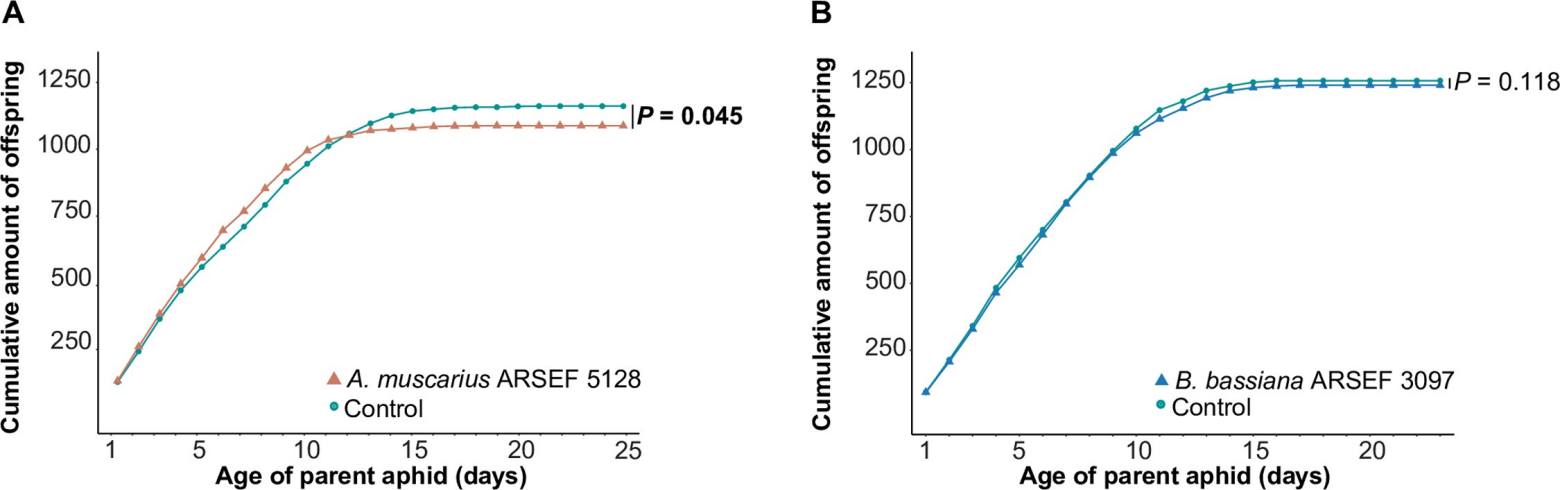
**Cumulative number of offspring produced by apterous *Myzus persicae* var. *nicotianae* when fed on sweet pepper plants inoculated with *Akanthomyces muscarius* ARSEF 5128 (orange) (A) or *Beauveria bassiana* ARSEF 3097 (blue) (B), compared to control plants (green) (20 biological replicates of one adult per plant).** Offspring was counted daily and removed after counting. *P* values refer to differences at the end of the experiment (Generalized Linear Mixed Model).

Nymphal mortality (*A*. *muscarius*: *P* = 0.376; *B*. *bassiana*: *P* = 0.100), nymphal development time (*A*. *muscarius*: *P* = 0.797; *B*. *bassiana*: *P* = 0.710) and adult morph ratio (*A*. *muscarius*: *P* = 0.105; *B*. *bassiana*: *P* = 0.940) were not significantly different between inoculated and non-inoculated plants (Table 2). Further, adult longevity was not significantly affected when aphids could feed on *B*. *bassiana*-inoculated plants compared to non-inoculated plants (*P* = 0.917). By contrast, aphids feeding on plants inoculated with *A*. *muscarius* had a significantly shorter adult life span compared to the control (*P* = 0.010). Adult aphids on control plants lived on average 19.6 ± 0.8 days, while aphids on plants inoculated with *A*. *muscarius* lived for 16.1 ± 1.0 days (Table 2). The total number of offspring produced by the 20 aphids tested did not differ between control plants and plants inoculated with *B*. *bassiana* (*P* = 0.118), but was significantly lower on *A*. *muscarius-*inoculated plants (54.1 ± 2.7) compared to control plants (60.3 ± 1.5) (*P =* 0.045) (Fig 4). Likewise, the reproductive period of aphids was shorter on plants inoculated with *A*. *muscarius*: the cumulative number of offspring levelled off after 12 days for *A*. *muscarius*-inoculated plants, whereas this was 15 days for non-inoculated plants (Fig 4). The intrinsic rate of natural population increase was not significantly different between inoculated and non-inoculated plants for both fungal species (*A*. *muscarius*: *P* = 0.771; *B*. *bassiana*: *P* = 0.876) (Table 2).

**Table 2:** Life history parameters^1^ of *Myzus persicae* var. *nicotianae* fed on sweet pepper plants inoculated with *Akanthomyces muscarius* ARSEF 5128 or *Beauveria bassiana* ARSEF 3097, compared to non-inoculated plants^2^.

Parameter	*A*. *muscarius* ARSEF 5128		*B*. *bassiana* ARSEF 3097	
	Inoculated	Non-inoculated	Inoculated	Non-inoculated
Nymphal mortality (%)	30.5 ± 4.0	26.0 ± 3.4	28.7 ± 2.5	33.9 ± 2.2
Development time (days)	6.2 ± 0.04	6.2 ± 0.04	7.0 ± 0.04	6.9 ± 0.04
Proportion winged morphs (%)	4.2 ± 1.4	1.6 ± 0.8	17.1 ± 2.6	17.1 ± 3.3
Adult longevity (days)	**16.1 ± 1.0**	**19.6 ± 0.8**	20.4 ± 1.2	20.8 ± 1.6
Total offspring per aphid	**54.1 ± 2.7**	**60.3 ± 1.5**	68.1 ± 1.8	62.9 ± 2.9
Intrinsic rate of natural population increase	0.43 ± 0.04	0.43 ± 0.02	0.40 ± 0.02	0.40 ± 0.03

^1^Average obtained from aphid experiments with twenty plants ± SEM (see main text for details).

^2^Values in bold are significantly different between fungus-inoculated and non-inoculated plants (*P* ≤ 0.05; ANOVA).

## Discussion

In the current study, effects of endophytic colonization by the entomopathogenic fungi *A*. *muscarius* ARSEF 5128 and *B*. *bassiana* ARSEF 3097 were examined on the behavioral response and life history of *M*. *persicae* var. *nicotianae* on sweet pepper plants. Both fungi were able to endophytically colonize aboveground tissues of sweet pepper following root inoculation, demonstrating that the fungi can translocate systemically throughout the plant system when roots were dipped in a conidia suspension, confirming previous studies [46–48]. Furthermore, none of the fungi were found on the outside of the leaves, confirming their endophytic presence. However, fungal strains were not detected by PCR in all plants investigated (on average in 78.2% of tested plants), possibly due to fungal abundances below the PCR detection limit or transient endophytic colonization, as reported previously for entomopathogenic fungi [10,49]. Indeed, there are reports of limited or no endophytic colonization of plants inoculated with entomopathogenic fungi despite positive effects on the plants (e.g. 19), particularly in non-sterile substrates [32], indicating that systemic establishment of the fungi as endophytes may not be the main cause of effects. In this study, plants were inoculated by root immersion, to obtain consistent endophytic colonization of the plants. However, as this method may be less adequate for field conditions, it is worth conducting future studies with alternative inoculation methods that may be more suitable for field or greenhouse conditions such as foliar spraying, seed inoculation, or substrate application [50].

Overall, artificial inoculation of sweet pepper plants with the tested fungi did not evoke strong changes in the behavioral response of aphids, which were only significantly attracted towards plants inoculated with *A*. *muscarius* ARSEF 5128. There are a few other studies that have evaluated the effect of endophytic entomopathogenic fungi on host plant selection by herbivores, but results are ambiguous. In line with our results, there are some studies that have shown that herbivores are attracted towards plants inoculated with endophytic entomopathogenic fungi [51,52]. For example, in a recent study, Fingu-Mabola et al. (2020) [51] showed that *M*. *persicae* preferred tobacco plants inoculated with *B*. *bassiana* or *Metarhizium acridum* over non-inoculated plants [51]. By contrast, in other studies, insect herbivores avoided plants inoculated with endophytic entomopathogenic fungi [23,28,29]. The mechanisms underlying these differences are still not clear, but are most likely due to differences in plant VOC profiles mediated by the fungi [53]. Host location (and selection) by herbivores is largely determined by plant VOC emissions, which are used as infochemicals to differentiate host plants from non-hosts, and to evaluate the suitability of different available hosts [54]. As such, changes in plant VOC composition or ratios between certain VOCs have been found to affect plant-insect interactions [55–57]. Our results revealed quantitative effects of fungal inoculation on certain VOCs emitted by sweet pepper plants. The elevated emission of β-pinene, indole and terpinolene by *A*. *muscarius-*inoculated plants may have caused aphid preference for these plants. Previous research using synthetic compounds has shown that β-pinene and indole can influence aphid behavior [58,59]. In addition, GC-EAG recordings from aphid antenna showed robust responses to indole [60]. Further research is needed to figure out which volatile component or combination of components is responsible for the observed changes in aphid behavior towards inoculated plants in our study. Although aphids preferred the odor of plants inoculated with *A*. *muscarius* ARSEF 5128, our results demonstrated small negative effects on some life history parameters. Adult longevity and fecundity were significantly reduced when aphids were feeding on *A*. *muscarius-*inoculated plants by 18% and 10%, respectively, in comparison with non-inoculated plants. These results are in line with previous findings showing negative effects of endophytic entomopathogenic fungi on life history parameters of herbivores [24,27,61,62]. For example, González-Mas et al. (2019) [24] found that endophytic colonization of melon plants with entomopathogenic fungi caused aphid mortality rates (*Aphis gossypii* Glover) ranging between 37.7% and 50.0% on endophytically colonized leaves, while this was only 13.7% in control plants [24]. Endophytic entomopathogenic fungi can infect plant-feeding insects when fungal propagules are ingested, resulting in mycosis [27,63]. However, mycosis of aphids that died after feeding on inoculated plants was not observed in this study. Furthermore, plating a number of dead aphids did not result in fungal outgrowth. The observed negative effects were most probably caused by changes in the composition and quantity of plant nutrients and/or defensive compounds within the plant [64,65]. Further metabolomic analyses of endophytically colonized plants and aphids feeding from these plants are required to confirm this scenario. Whereas adult longevity and reproduction were affected by endophytic colonization, we did not find any influence of endophytic entomopathogenic fungi on nymphal mortality, nymphal development and formation of winged morphs, which often indicates the presence of poor food quality or other stress factors [66]. There was, however, a substantial difference in the number of winged aphids between both fungi. However, as no differences were observed with the corresponding controls, these differences were most probably due to different environmental conditions under which the experiments were performed. Environmental parameters like temperature are known to affect population growth and the proportion of winged aphid morphs [67,68]. Nevertheless, Jaber and Araj (2018) [38] reported negative effects of endophytic entomopathogenic fungi on these life history parameters for *M*. *persicae* [38]. The difference between this study and our study may be explained by the fungal strains used, the host cultivars or the aphid lineages, all of which are known to play a major role in the interactions between fungi, plants and herbivores [69]. Furthermore, it has to be noted that our experiments were performed on only one generation of aphids, while effects may become more pronounced over multiple generations [38,70].

## Conclusion

Our results indicate that endophytic entomopathogenic fungi have the potential to alter the olfactory behavior and performance of pest insects, but the effects observed in our study were small and depend on the strain used. Root inoculation of sweet pepper plants with *A*. *muscarius* ARSEF 5128 altered the plants VOC profile and elicited a positive olfactory response of *M*. *persicae* var. *nicotianae* towards sweet pepper plants, but significantly reduced aphid survival and fecundity on these plants. Although statistically significant, reduction in fecundity was very small, and aphids had a similar intrinsic rate of population increase compared to non-inoculated plants, indicating that effects on the population level will be limited. Sweet pepper plants inoculated with *B*. *bassiana* ARSEF 3097 did not elicit a significant behavioral response and inoculation with this strain did also not affect the investigated life history traits.

## Supporting information

S1 Fig Set up (A) and schematic overview (B) of the two-choice arena assay in which 10 apterous Myzus persicae var. nicotianae were given the choice between sweet pepper plants inoculated with Akanthomyces muscarius ARSEF 5128 or Beauveria bassiana ARSEF 3097 and control plants. Plants were laid on their side, and 4 cm of the fifth true leaf of a non-inoculated control plant and the fifth true leaf of an inoculated plant were fed through a slit in the short sides of a rectangular Petri dish (9 cm × 12.5 cm × 1.5 cm). Leaves were fixed with a droplet of agar on their adaxial side at the bottom of the plate. Afterwards, the plate was flipped so that the leaves regained their natural position, and the aphids were allowed to walk on the abaxial side of the leaves. For each test, ten apterous adults, starved for 1 h, were released in the middle of the arena, after which the arena was sealed with parafilm to prevent aphids from escaping.
(DOCX)

S1 Table Results of PCR detection of inoculated fungi in sweet pepper plants^1^. (DOCX)
