# Supplementary material for: Impact of endophytic colonization by entomopathogenic fungi on the behavior and life history of the tobacco peach aphid Myzus persicae var. nicotianae
Source: PLoS One. 2022 Sep 6;17(9):e0273791. doi: 10.1371/journal.pone.0273791 (PMC9447930; doi:10.1371/journal.pone.0273791)
Supplement: S1 Fig — Set up (A) and schematic overview (B) of the two-choice arena assay in which 10 apterous Myzus persicae var. nicotianae were given the choice between sweet pepper plants inoculated with Akanthomyces muscarius ARSEF 5128 or Beauveria bassiana ARSEF 3097 and control plants. Plants were laid on their side, and 4 cm of the fifth true leaf of a non-inoculated control plant and the fifth true leaf of an inoculated plant were fed through a slit in the short sides of a rectangular Petri dish (9 cm × 12.5 cm × 1.5 cm). Leaves were fixed with a droplet of agar on their adaxial side at the bottom of the plate. Afterwards, the plate was flipped so that the leaves regained their natural position, and the aphids were allowed to walk on the abaxial side of the leaves. For each test, ten apterous adults, starved for 1 h, were released in the middle of the arena, after which the arena was sealed with parafilm to prevent aphids from escaping. (DOCX) [file pone.0273791.s001.docx]

**B**

A


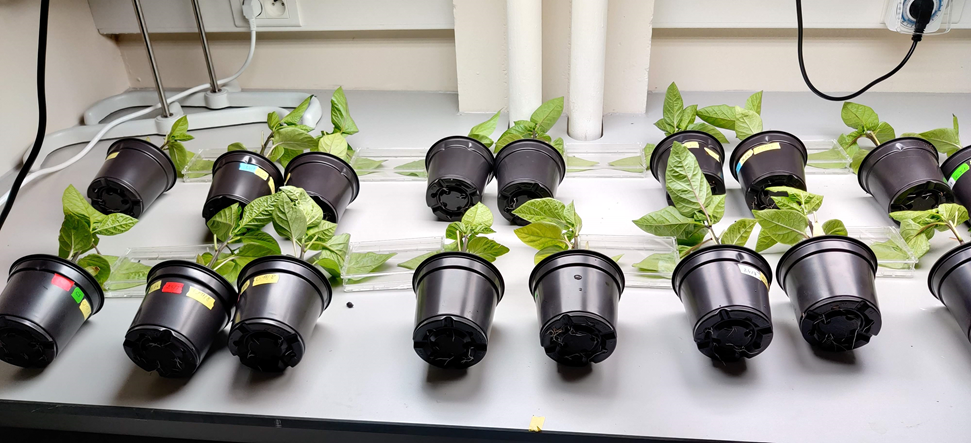


**A**


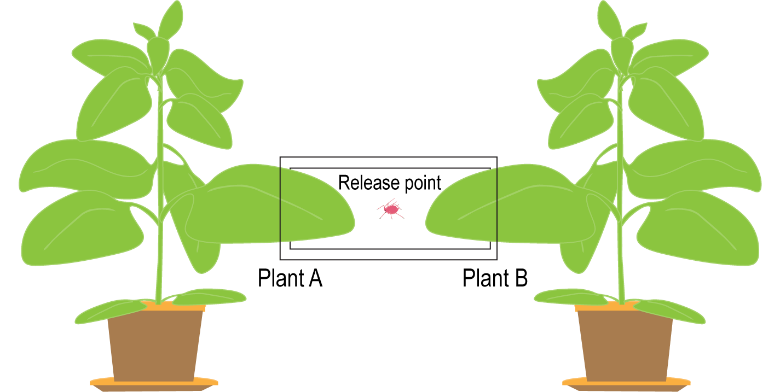


**Figure S1. Set up (A) and schematic overview (B) of the two-choice arena assay in which 10 apterous *Myzus persicae* var. *nicotianae* were given the choice between sweet pepper plants inoculated with *Akanthomyces muscarius* ARSEF 5128 or *Beauveria bassiana* ARSEF 3097 and control plants.** Plants were laid on their side, and 4 cm of the fifth true leaf of a non-inoculated control plant and the fifth true leaf of an inoculated plant were fed through a slit in the short sides of a rectangular Petri dish (9 cm × 12.5 cm × 1.5 cm). Leaves were fixed with a droplet of agar on their adaxial side at the bottom of the plate. Afterwards, the plate was flipped so that the leaves regained their natural position, and the aphids were allowed to walk on the abaxial side of the leaves. For each test, ten apterous adults, starved for 1 h, were released in the middle of the arena, after which the arena was sealed with parafilm to prevent aphids from escaping.
