# Supplementary material for: Impact of endophytic colonization by entomopathogenic fungi on the behavior and life history of the tobacco peach aphid Myzus persicae var. nicotianae
Source: PLoS One. 2022 Sep 6;17(9):e0273791. doi: 10.1371/journal.pone.0273791 (PMC9447930; doi:10.1371/journal.pone.0273791)
Supplement: S1 Table — (DOCX) [file pone.0273791.s002.docx]

**S1 Table. Results of PCR detection of inoculated fungi in sweet pepper plants^1^.**

| **Experiment** | **Treatment** | ***A. muscarius* ARSEF 5128** | ***B. bassiana* ARSEF 3097** |
| --- | --- | --- | --- |
| **Two-choice Y-tube assay** | *A. muscarius-*inoculated | 10/10 | 0/10 |
|  | *B. bassiana-*inoculated | 0/10 | 8/10 |
|  | Non-inoculated | 0/10 | 0/10 |
| **Two-choice arena assay** | *A. muscarius-*inoculated | 9/10 | 0/10 |
|  | *B. bassiana-*inoculated | 0/10 | 5/10 |
|  | Non-inoculated | 0/10 | 0/10 |
| **VOC analysis** | *A. muscarius-*inoculated | 5/9 | 0/9 |
|  | *B. bassiana-*inoculated | 0/9 | 7/9 |
|  | Non-inoculated | 0/9 | 0/9 |
| **Life history** | *A. muscarius-*inoculated | 7/10 | 0/10 |
|  | *B. bassiana-*inoculated | 0/10 | 10/10 |
|  | Non-inoculated | 0/10 | 0/10 |

^1^Samples of the fifth true leaf were taken and analyzed by PCR. A number of amplicons were sequenced, confirming the identity of the fungus. Results are expressed as the ratio between the number of positive samples and the total number of samples tested.
